# Periodontal health and pregnancy outcomes: Time to deliver

**DOI:** 10.1111/aogs.14548

**Published:** 2023-05-22

**Authors:** Nagihan Bostanci

**Affiliations:** ^1^ Section of Oral Health and Periodontology, Division of Oral Diseases, Department of Dental Medicine Karolinska Institutet Stockholm Sweden

## 1 ORAL HEALTH AND PREGNANCY

Oral diseases affect over 3.5 billion people worldwide and entail substantial expenses for individuals and society. Periodontal diseases (gingivitis and periodontitis) are frequently neglected, and yet represent the sixth most prevalent non‐communicable oral diseases affecting 796 million people worldwide according to the Global Burden of Disease study.^1^ These diseases are typically initiated by the accumulation of dental plaque on the tooth and root surfaces.^2^ Evidently, there are many connections between systemic and periodontal health. Changes in hormones such as those that occur during puberty, menstruation, pregnancy, and menopause, or with the intake of hormonal supplements, can all alter the periodontal health of women.^3^

In the current issue, Thomas et al. demonstrate that almost half of the pregnant women in their first trimester exhibited poor periodontal health, while those who developed periodontitis were more prone to develop gestational diabetes during their pregnancy.^4^ These findings are in line with existing evidence alluding to periodontal diseases as systemic stressors during pregnancy, that are also associated with adverse pregnancy outcomes (APOs). Such considerations have nonetheless received little attention thus far in everyday clinical practice, due to several existing barriers.

Importantly, up to one third of the pregnant women were unaware of their existing periodontal condition, as symptoms of gingival inflammation remained concealed. This seems to be a common pattern across the world as periodontitis is considered a “silent epidemic”, that may start and worsen with little or no pain. If left untreated, not only will periodontal diseases destroy the tooth supporting tissues, but they may further constitute a risk factor to several life‐threatening affections such as cardiovascular diseases, diabetes mellitus II, as well as to APOs. “Periodontal Health for a Better Life” has been emphasized by several health associations including the European Federation of Periodontology and World Health Organization among the few.

The progesterone and estrogen levels are known to peak during the second and third trimester of pregnancy, when one half of women with pre‐existing gingival inflammation are documented to exhibit significant exacerbation of bleeding in their gums in combination with changes in specific oral taxa, such as *Porphyromonas gingivalis* and *Prevotella melaninogenica*.^5^ Driven by these findings, the current body of literature is mainly focused on the second and third pregnancy trimesters, somewhat overlooking the first trimester, which now appears to be the tipping point to implement efficient prevention strategies. The systematic reviews show that the percentage of pregnant women with periodontitis varies significantly from 16% to 67% and seems to be dependent on the time of the clinical examination, the type of periodontal examination performed (partial‐mouth vs full‐mouth) as well as on the case definition of periodontitis.^6^

## 2 COMMON PERIODONTAL PROBLEMS IN PREGNANCY

The reproductive age of a woman's lifetime, spans from menarche until menopause and is characterized by major changes in circulating hormone levels, frequently accompanied by a worsened oral health, altered saliva composition and considerable changes in structure (increased vascular permeability), cellular and humoral immune responses of the tooth supporting periodontal tissues (periodontium). Gingival tissues are indeed known to express estrogen‐specific receptors and hence to represent an estrogen‐sensitive tissue.

Over 150 years, the gingival and periodontal inflammation observed in pregnant women were considered to be separate conditions from that of plaque‐induced gingivitis.^7^ Pregnancy‐associated gingivitis is the most common reversible condition during pregnancy, with a prevalence ranging from 30% to 100%. Pregnancy‐associated gingivitis is typically characterized by a red swollen gingiva, that readily bleeds when one brushes or flosses. Although it is rare and benign, 0.5%–10% of pregnant women also experience a localized gingival enlargement namely, pregnancy‐associated pyogenic granuloma or epulis gravidarum mainly due to the local accumulation of dental calculus. In most cases, these changes are transient and come to resolution during the post‐partum period, without loss of periodontal support. Whereas one cannot assert that pregnancy exacerbates pre‐existing periodontitis, or that multiple pregnancies result in risk accumulation, women with pre‐existing disease can reduce the risk of recurrence during pregnancy through proper oral hygiene and essential periodontal care as needed (i.e., scaling and root planning, known as deep cleaning).

## 3 ASSOCIATION BETWEEN MATERNAL PERIODONTITIS AND ADVERSE PREGNANCY OUTCOMES

**FIGURE 1:**
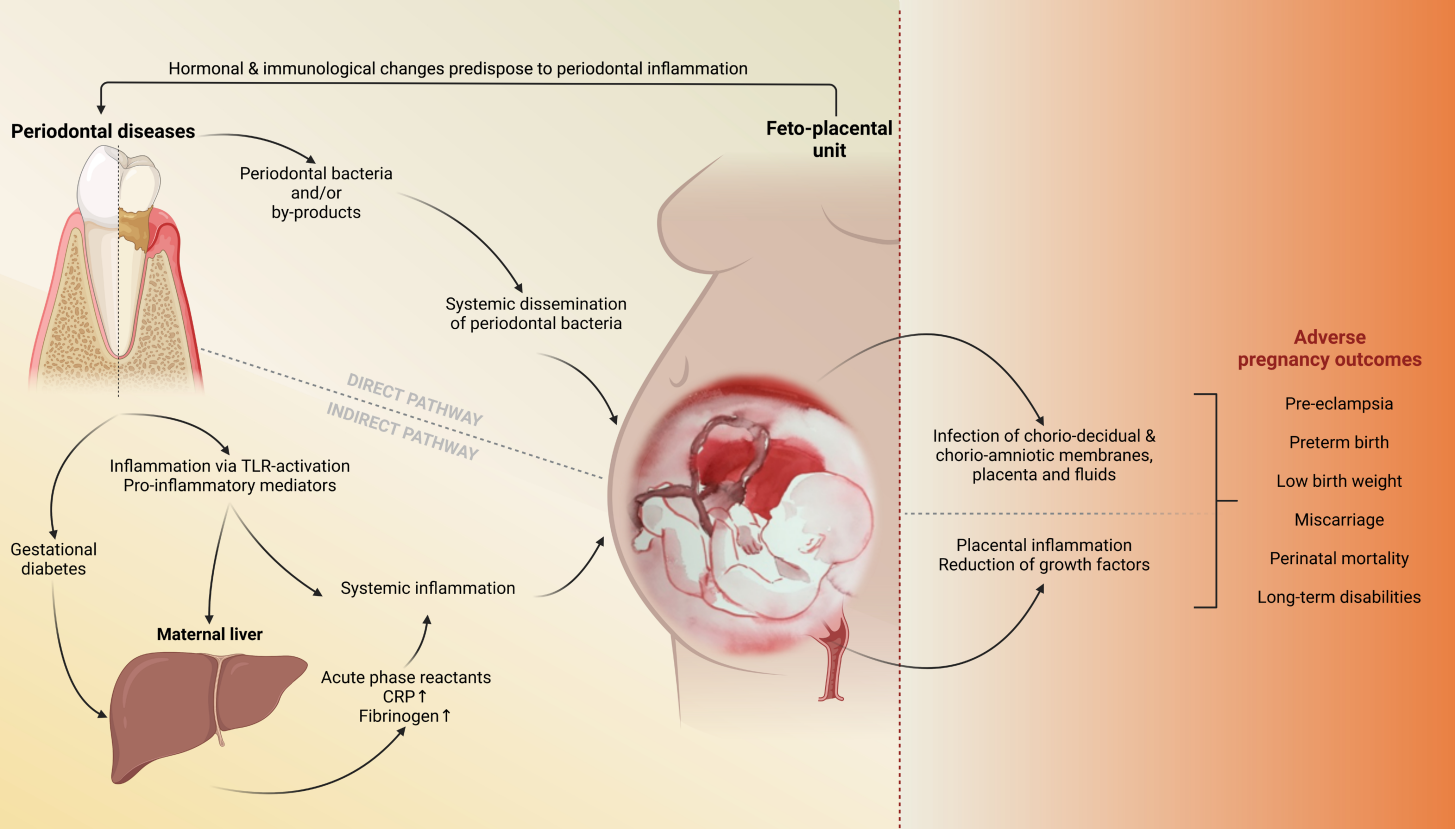
Periodontitis and adverse pregnancy outcomes. Proposed model of association between periodontitis and adverse pregnancy outcomes. The “direct pathway” mechanism purports that periodontal bacteria and their toxins may translocate from the subgingival biofilm and disseminate via the bloodstream to colonize membranes and fluids of the feto‐placental unit. The “indirect pathway” purports that the periodontal inflammation leads to an increase of circulatory inflammatory mediators stemming directly from periodontal tissues, as well as from a systemic induction of acute phase reactants. The “direct” and “indirect” pathways may occur simultaneously and ultimately induce imbalances in thrombogenic factors, impaired placental development, membrane rupture or uterus contraction. Such conditions may further cause severe adverse pregnancy outcomes. CRP, C‐reactive protein; TLR, Toll‐like receptor. Designed using BioRender.com.

Periodontitis in the carrying mother has been associated with several APOs including the delivery of preterm and/or low birthweight babies, pre‐eclampsia, or gestational diabetes when all other cofounding risk factors have been taken into consideration (Figure 1). However, the strength of the association found between periodontitis and APOs varies between studies, and the underlying mechanism remains unclear. Reportedly, in the present cohort, maternal periodontitis is independently associated with gestational diabetes mellitus (GDM), yet directly associated with birthweight or gestational age. The authors also highlighted the importance of more robust prospective study designs to substantiate these findings. Whereas the etiology of GDM is not fully elucidated, a low‐grade systemic inflammation caused by periodontitis that would affect insulin signaling and glucose metabolism is suspected. There is a limited number of studies that suggest a potential association between maternal periodontitis and GDM. A more recent systematic review and meta‐analysis of 10 studies suggests that periodontitis is associated with two‐fold increased odds of GDM compared to women without periodontitis after adjusting for potential confounders.^8^ Establishing periodontitis as a risk factor for GDM may provide new public health intervention strategies for the prevention of mother's GDM and its adverse effects on the infant.

In the early 1990s, the biological plausibility of this association has been attributed to the microbial translocation into the peripheral bloodstream via disruption of the gingival epithelial barrier, and systemic trafficking of virulence factors during pregnancy to the intrauterine environment where they cause inflammatory and immune responses affecting the feto‐placental unit in experimental models.^9^ Furthermore, typical periodontal taxa such as *P. gingivalis* or *Fusobacterium nucleatum* have been retrieved from amniotic fluid samples collected in women with preterm labor and intact membranes. Nonetheless, whether the human fetus and the prenatal intrauterine environment are stably colonized by microbial communities in a healthy pregnancy remains a subject of debate. The most recent evidence does not support the presence of an “in‐utero microbiome” at term‐pregnancies.^10^ Additionally, large studies investigating several oral bacteria found no associations between the presence or abundance of specific periodontal pathogens and APOs. However, it should be noted that dental plaque in the form of multispecies oral biofilms is necessary but not sufficient to cause periodontitis alone. Rather, periodontal tissue destruction results from host immunoinflammatory processes triggered by dysbiotic shifts that occur within a complex microbial plaque biofilm. The association between periodontitis and APOs may thus reflect the maternal immune‐inflammatory trait as seen in genitourinary tract infections, which can elicit low birthweight and spontaneous abortions in humans but rarely involve direct bacterial invasion of the fetal‐placental unit. Supportive of this hypothesis, earlier work suggested that the risk for APOs may be associated with a less robust antibody response to specific periodontal pathogens during the second trimester.^11^ Because such diminished antibody response may be a shared factor with the onset of periodontitis, the disease could be an early sign to take into consideration for the prevention of APOs. In the spotlight of the findings of Thomas et al. that periodontitis is widespread in carrying women during the first trimester, it would be surprising if periodontal intervention carried out during the second trimester of pregnancy did improve APOs. It is important to examine this temporal relation using prospective studies that provide periodontal examination and, if needed, periodontal interventions for all women who are planning to conceive.

## 4 PERIODONTAL TREATMENT IS SAFE AND EFFECTIVE DURING PREGNANCY

One main oral health goal during pregnancy should be to avoid excessive dental plaque accumulation by enhancing oral hygiene and regular check‐ups with either an oral or prenatal healthcare professional. The achievement of an adequate periodontal health in pregnant women has in the past been hindered by myths surrounding the safety of dental/periodontal care during pregnancy. Thomas et al. reports that only 32.9% of the 407 pregnant women included in the larger obstetrical risk screening study were available for oral health screening and some dropped out due to dentist phobia. As far as their oral health was concerned, 41.3% had not been to the dentist at least one year prior to pregnancy. These findings are consistent with existing reports showing that there is low attendance to dental services among pregnant women due to a lack of oral health education, difficulties of access, along with dentist fear and concerns for the safety of the fetus. Specific dental plaque removal approaches together with professional oral health interventions, have been shown to be safe when implemented during the second trimester of pregnancy and efficient both in reducing gingival inflammation and enabling periodontal health to be maintained during pregnancy.^12^ Pregnancy is a time when women may be more susceptible to learn about health improvement strategies, and eager to implement them into their daily life. Therefore, oral health and prenatal health professionals should frequently communicate on maternal‐oral relations with women in their fertility years and emphasize on the possible benefits of pre‐pregnancy interventions (at home or office) for the establishment of healthy periodontal conditions. Promotion of periodontal health education and better integration of oral health into prenatal health care, particularly among vulnerable women groups, may be beneficial to health and well‐being of the mother and child.
